# Tirzepatide and the NHS: are we creating a two-tier obesity treatment system?

**DOI:** 10.3399/BJGP.2025.0610

**Published:** 2025-10-27

**Authors:** Laurence J Dobbie, Mariam Molokhia, John PH Wilding, Tricia M-M Tan, Barbara McGowan

**Affiliations:** 1 School of Life Course & Population Sciences, King’s College London, London, UK; 2 Department of Diabetes and Endocrinology, Guy's and St Thomas' NHS Foundation Trust, London, UK; 3 Obesity Management Collaborative UK, London, UK; 4 Department of Cardiovascular and Metabolic Medicine, Institute of Life Course and Medical Sciences, University of Liverpool, Liverpool, UK; 5 Department of Metabolism, Digestion and Reproduction, Imperial College London, London, UK; 6 Diabetes and Nutritional Sciences, King's College London, London, UK

Obesity is a global health crisis and is associated with significant health risks, including cardiovascular disease (CVD), type 2 diabetes (T2D), and cancer. The NHS’s 12-year phased rollout of tirzepatide (Mounjaro^®^) represents a paradox in obesity care; while creating options for effective obesity care, its implementation risks exacerbating already serious health inequalities. Analysis by IQVIA from March 2025 estimates over 1.5 million UK residents access GLP-1 receptor agonists (GLP-1RA) privately, but the NHS is planning to treat only 220 000 patients in the first 3 years; creating a two-tier treatment system that disadvantages our most vulnerable patients. The social determinants of health significantly impact obesity: prevalence in adults is nearly double in the most deprived areas (36%) compared to the least deprived (20%).^1^ The Phase 1 criteria for NHS prescription of tirzepatide, BMI ≥40 kg/m² plus four of five specific comorbidities (T2D or pre-diabetes, hypertension, dyslipidaemia, established CVD, obstructive sleep apnoea [OSA]), is arguably the strictest medication rollout in NHS history. Although this approach does allow some access to transformative obesity management medications on the NHS, unlike many European countries, rationing therapy to a small subset of patients creates substantial diagnostic and treatment barriers, particularly for vulnerable groups.

Eligibility criteria that require multiple diagnosed comorbidities are likely to exacerbate inequalities, because qualifying conditions are underdiagnosed among people with serious mental illness (SMI), those from lower socioeconomic backgrounds, women, and people from minority ethnic groups. E-ECHOES, a large UK-based heart health study in minority ethnic communities, reported undiagnosed hypertension in 37% of South Asians and 20.8% of African-Caribbeans.^2^ Underdiagnosis of CVD and T2D is higher in deprived areas.^3^ Health survey for England data shows higher rates of undiagnosed T2D in South Asian and Black adults than in White British adults, even after accounting for socioeconomic status; lower educational attainment also independently increases the rates.^4^ Women experience systematic underdiagnosis of CVD due to non-specific symptom presentations, absence of sex-specific criteria and biases in care and research. This diagnostic gap in CVD stems from poorer CVD screening and uptake in low socioeconomic groups and women, socioeconomic deprivations influence on GP cardiology referral rates, lower NHS health check uptake among minority ethnic groups, and limited access to CVD diagnostics.^5,6^

*“At present, more than 1.5 million people access GLP-1RAs privately, while NHS provision remains tightly rationed, creating a two-tier obesity treatment system.”*

Patients with SMI (for example schizophrenia and bipolar affective disorder) have a 15–20-year lower life expectancy which is driven primarily by CVD. Although obesity-related conditions are common, 27.7% of patients with SMI are excluded from annual physical-health checks at least once.^7^ A meta-analysis reports 39% less CVD screening and almost 50% lower rates of invasive coronary procedures in schizophrenia-spectrum disorders.^8^ Underdiagnosis in SMI may also affect hypertension, valvular heart disease, arrhythmias and obstructive sleep apnoea (OSA), limiting eligibility for tirzepatide under current criteria.^7–9^

Although NICE guidance makes an allowance for lower BMI thresholds for minority groups, Phase 1 criteria may still exclude these groups because of barriers to healthcare access and provider biases. People with SMI are similarly disadvantaged: despite higher T2D prevalence, they receive inadequate cardiometabolic monitoring (BMI, blood pressure, blood glucose, cholesterol).^9^ These inequalities are worsened by regional variation; referral rates to weight management services are lower in rural GP practices and there is a five-fold difference between the highest and lowest performing regions of England (West Midlands: 5.40%, East of England: 1.04%).^10^ Freedom-of-information analysis also indicates significant regional inequality in tirzepatide roll-out, as of August 2025, only 8 of 42 integrated care boards (ICBs) provide tirzepatide for obesity, with several planning caps on first-year numbers.

The wrap-around nutritional, functional and psychological care may also widen inequalities. Analysis from the NHS Diabetes Prevention Programme showed that those from minority ethnic groups and lower socioeconomic backgrounds lose less weight and experience less HbA1c reduction.^11^ The nationally commissioned wrap-around care, Behavioural Support for Obesity Prescribing (BSOP), uses a similar model; unless delivery is culturally adapted, responsive to social needs (for example, food insecurity), accommodating of medical complexity, and has adaptions for people with SMI, it risks exacerbating inequalities in therapeutic outcomes.

Excluding the Society for Endocrinology/Obesity Management Collaborative-UK (SFE/OMC-UK) prioritisation criteria represents a missed opportunity. This was developed to guide GLP-1RA roll-out and facilitate patient prioritisation (Box 1). The SFE/OMC-UK have identified clinical contexts, not recognised in the current Phase 1 criteria, where weight loss confers particular benefits: improved cancer outcomes, expedited access to time-sensitive surgery, or meeting eligibility thresholds for fertility services.^12^ Knee replacement surgery exemplifies this challenge: excess weight precludes surgical eligibility, with delays worsening pain, disability, and psychological well-being, and physical disability contributing towards further weight gain. Furthermore, the absence of efficacy data among minority ethnic groups undermines equitable obesity care. GLP-1RA trials under-recruit minority ethnic participants, with London-based data reporting lower efficacy and retention among Black populations.^13^

**Box 1.** Society for Endocrinology and Obesity Management Collaborative-UK (SFE/OMC-UK) Semaglutide Criteria and the NHS England Tirzepatide Phased Roll-out^a^,

Medication	Phase 1 Eligibility Criteria (Summary)
Semaglutide 2.4 mg^b^(SFE/OMC‑UK Phase 1 Criteria)^12^	Patients with the most urgent medical needs, including those requiring weight loss to improve outcomes or eligibility for time-critical treatments (for example, cancer treatments, organ transplantation, idiopathic intracranial hypertension with visual compromise), to enable time-sensitive surgery (where high BMI is the primary barrier to surgery and weight loss would be beneficial) or access to fertility treatments where weight loss would be beneficial, or those with severe obesity-related respiratory disease (for example, severe obstructive sleep apnoea or obesity-hypoventilation syndrome).
Tirzepatide 15 mg^b^(NHS England Cohort I)^14^	Eligibility limited to adults with BMI ≥40 kg/m²^c^ and at least four of five specified comorbidities (type 2 diabetes, hypertension, dyslipidaemia,established cardiovascular disease, or obstructive sleep apnoea).

a Note this table does not include the full NICE tirzepatide funding variation criteria or SFE/OMC-UK phase 1 criteria and phase 2 to 4 criteria due to space constraints. Please see the NICE guideline and SFE/OMC-UK position statement for further details.

b All prescriptions require wrap-around care, including a reduced-calorie diet and increased physical activity within a comprehensive obesity-management plan. Contraception is required while taking these medications ahead of planned assisted conception.^12^

c Apply ethnicity-adjusted BMI thresholds (typically −2.5 kg/m² for minority ethnic groups).

A BMI ≥50 kg/m² is associated with severe functional disability. Making BMI ≥50 kg/m² (≥47.5 kg/m² for minority ethnic groups) an automatic qualifier, irrespective of complications, would recognise obesity as a disease and improve access. This threshold could then be reduced gradually to BMI ≥40 kg/m² (≥37.5 kg/m² for minority ethnic groups), better capturing minority ethnic, deprived, and SMI populations who are at higher risk of undiagnosed obesity-related complications. The BMI ≥50 kg/m² (≥47.5 kg/m² for minority ethnic groups) aligns with bariatric-surgery guidance, which permits direct referral without completion of a Tier 3 specialist weight management programme.

## Recommendations for improving equity of access in the NHS

1. Adapting thresholds for qualification to ≥50 kg/m² (≥47.5 kg/m² for minority ethnic groups) without complication, with phased reduction of thresholds to BMI ≥40 kg/m² (≥37.5 kg/m² for minority ethnic groups) by 2030.
2. Pathways taking into account ethnicity and considering underdiagnosis and lower BMI thresholds.
3. SMI-specific pathways considering underdiagnosis and the unique challenges faced by this population.
4. Accelerating the rollout timeline.
5. Integrating digital health services to help overcome regional inequalities and geographical barriers to care.
6. Including current SFE/OMC-UK criteria for eligibility.

Equitable access to pharmacotherapy must be matched by upstream public-health measures addressing dietary policy, food insecurity, and urban planning. At present, more than 1.5 million people access GLP-1RAs privately, while NHS provision remains tightly rationed, creating a two-tier obesity treatment system. The combination of strict phase 1 eligibility requiring multiple qualifying comorbidities, together with underdiagnosis among minority ethnic groups, women, and people with SMI, creates avoidable barriers to care. These are compounded by geographical variation and caps on patient numbers during ICB roll-out. The wrap-around care may also widen or narrow inequalities depending on whether delivery is culturally adapted, addresses social needs, accommodates medical complexity and has adaptions for people with SMI. As new obesity management medications become available, policy should prioritise those with the highest clinical need, particularly those with severe obesity. Without action, these inequalities will persist and worsen for the next generation.
